# Understanding Bullying and Cyberbullying Through an Ecological Systems Framework: the Value of Qualitative Interviewing in a Mixed Methods Approach

**DOI:** 10.1007/s42380-022-00126-w

**Published:** 2022-05-10

**Authors:** Faye Mishna, Arija Birze, Andrea Greenblatt

**Affiliations:** Factor-Inwentash Faculty of Social Work, University of Toronto, 246 Bloor Street West, Toronto, ON M5S 1V4 Canada

**Keywords:** Qualitative research benefits, Mixed methods research, Bullying and cyberbullying, Voices of children and youth

## Abstract

Recognized as complex and relational, researchers endorse a systems/social-ecological framework in examining bullying and cyberbullying. According to this framework, bullying and cyberbullying are examined across the nested social contexts in which youth live—encompassing individual features; relationships including family, peers, and educators; and ecological conditions such as digital technology. Qualitative inquiry of bullying and cyberbullying provides a research methodology capable of bringing to the fore salient discourses such as dominant social norms and otherwise invisible nuances such as motivations and dilemmas, which might not be accessed through quantitative studies. Through use of a longitudinal and multi-perspective mixed methods study, the purpose of the current paper is to demonstrate the ways qualitative interviews contextualize quantitative findings and to present novel discussion of how qualitative interviews explain and enrich the quantitative findings. The following thematic areas emerged and are discussed: augmenting quantitative findings through qualitative interviews, contextualizing new or rapidly evolving areas of research, capturing nuances and complexity of perspectives, and providing moments for self-reflection and opportunities for learning.

## Introduction

Bullying and cyberbullying are increasingly recognized as complex phenomena that are considered relationship problems (Mishna et al., 2021a; Pepler et al., 2010; Pepler, 2006; Spears et al., 2009). Appreciating that individuals are embedded in and both shape and are shaped by systems of relationships (Bronfenbrenner & Morris, 2007), researchers often endorse an ecological systems framework as paramount and comprehensive in examining bullying and cyberbullying phenomena^1^ (Espelage, 2014; Newman et al., 2018; Thornberg, 2015, 2018). According to this approach, individuals are embedded in and affected by interconnected and layered systems (Bronfenbrenner, 1979, 1992). Children’s social-emotional development at school is consequently shaped not only by children’s relationships with their teachers and peers, but also by the interconnections between these relationships and the other layers of social ecology, all of which are considered to contribute to social behavioral patterns (O'Moore & Minton, 2005). Bullying and cyberbullying are examined across the nested social contexts in which youth live—encompassing individual features, peer relationships, school, family, and ecological climate such as societal norms and conditions as well as online technology (Cross et al., 2015; Johnson, 2010; Nesi et al., 2018). An ecological systems framework is considered an overarching approach that many theories complement and within which they fit (Bauman & Yoon, 2014).

The purpose of the current paper is to demonstrate the contributions of qualitative research in understanding the phenomena of bullying and cyberbullying and enriching and complementing the findings of quantitative methodology (Creswell & Creswell, 2018). Qualitative inquiry of bullying and cyberbullying provides a research methodology capable of bringing to the fore salient discourses and otherwise invisible nuances that might not be accessed through quantitative studies (Dennehy et al., 2020).

There are advantages to utilizing mixed methods in conducting research on various topics including cyberbullying (Creswell & Creswell, 2018). When engaging with complex phenomena such as cyberbullying, conceptual and methodological multiplicity offers distinct insights into research questions (McKim, 2017; Thornberg, 2011). When quantitative and qualitative research are used in combination, it is possible to obtain deeper as well as more comprehensive and accurate understanding of young people’s experiences, which increases the likelihood of informing strategies and responses that can effectively address the needs of children and adolescents (Crivello et al., 2009; Darbyshire et al., 2005; Fevre et al., 2010). The quality of findings may be strengthened when researchers use mixed methods because the data are triangulated (Crivello et al., 2009). Data generated through diverse research methods can both complement and contradict each other, which offers an opportunity to better understand the complexities of cyberbullying (Hemming, 2008). While quantitative approaches strive for objectivity by examining general concepts, such as cyberbullying, and parceling those concepts into specific, concrete, and understandable behaviors (Fevre et al., 2010), qualitative interviews give voice to children and youth, enabling them to express their thoughts and feelings about themselves, their relationships, environments, and the world in which they live (Mishna et al., 2004; Chaumba, 2013; Dennehy et al., 2020; Patton et al., 2017).

Through qualitative interviewing, we can step outside the bounds of adult thinking, gaining insights and discovering unanticipated differences in the perceptions of adults and children (Dennehy et al., 2020; O’Farrelly, 2021). To understand the phenomena of bullying and cyberbullying and inform effective prevention and intervention strategies, it is argued, children’s own views, “are at the heart of these efforts” (O’Farrelly, 2021, p. 43). Thus, we present findings from the qualitative component of our Canadian federally funded mixed methods longitudinal study on cyberbullying from the perspectives of school-aged youth and their parents and teachers, entitled *Motivations for Cyber Bullying: A Longitudinal and Multi-Perspective Inquiry*^2^ (Mishna et al., 2016).

### Background Study Description

The objectives of our longitudinal mixed methods study were to (1) explore youth experiences and perspectives and their parents’ and teachers’ conceptions of cyberbullying; (2) explore how youth and adults view the underlying motivations for cyberbullying; (3) document the prevalence rates of cyberbullying victimization, witnessing, and perpetration; (4) identify risk and protective factors for cyberbullying involvement; and (5) explore social, mental health, and health consequences of cyberbullying among children and youth aged 9 to 18 (grades 4, 7, and 10) over 3 years.

## Methods

In addressing the objectives, we use an explanatory sequential mixed methods design (Creswell & Creswell, 2018). The study comprised a 2-phase data collection approach in which we first collected the quantitative data and then used findings from the first phase to design and plan the qualitative data phase. The quantitative findings informed both our selection of interview participants and the focus of questions we wanted to explore further in the interviews. The overall intent of the qualitative interviews was to enrich and expand upon the quantitative findings and perhaps generate and explore similarities and contradictions (Creswell & Creswell, 2018). In the current paper, we briefly review key quantitative findings. We then discuss the qualitative findings and how they provide more depth and insight and demonstrate the complexities of bullying and cyberbullying motivations, behaviors, and attitudes. In so doing, we present novel discussions of how the qualitative interviews augment the quantitative findings.

### Participants

Three participant groups were included in the baseline study sample: (1) students in 4th (*n* = 160), 7th (*n* = 243), and 10th (*n* = 267) grades; (2) their teachers (*n* = 103); and (3) their parents (*n* = 246). A stratified random sampling strategy was utilized to select participants. First, a random sample of 19 schools was drawn from one of the largest school boards in North America. Schools were stratified into three categories of need (low, medium, and high) based on an index developed by the school board that ranked schools on external challenges to student achievement (Toronto District School Board, 2014). This stratification ensured representation of ethno-cultural and socioeconomic diversity—factors that potentially impact access to Information and Communication Technologies (ICTs), experiences of cyberbullying, and the manifestation of negative outcomes (Lenhart et al., 2015; Steeves & Marx, 2014). In year 3 of the study, 10 additional schools were recruited for participation to follow those students transitioning from elementary/middle school to middle/secondary school. A total of 29 schools participated in the study. All students in the selected grades at the original participating schools were invited to participate, as were their parents and teachers.

Participating students and their parents provided data in all 3 years of the study, while matching teachers provided data in year 1 only (as student participants’ teachers changed each year). All three participant groups completed quantitative questionnaire packages, and a sub-sample of each group participated in individual interviews. Quantitative data were collected from students and parents in each year of the study, while qualitative data were collected during years 1 and 3, to allow for enough time to elapse for changes in perceptions of cyberbullying to emerge.

#### **Quantitative Measures and Analysis**

In year 1, students completed a 45–60-min quantitative questionnaire package in the school setting, while parents completed a questionnaire package by mail. Questionnaires for teachers, which took approximately 45–60 min to complete, were administered in the participating schools. This study utilized several quantitative measures, including standardized measures and measures developed specifically for the study. Student, parent, and teacher surveys obtained information related to experiences with bullying/cyberbullying (Mishna et al., 2012; Unpublished Survey), socio-demographics, and Information and Communication Technology (ICT) use. Standardized measures assessing student mental health, health, social, and behavioral issues included Child Behavior Check List (Achenbach, 2001a), Teacher Report Form (Achenbach, 2001b), Youth Self Report Form (Achenbach, 2001c), Self-Perception Profile for Children (Harter, 1985b), Self-Perception Profile for Adolescents (Harter, 2012), Social Support Scale for Children (Harter, 1985a), and Social Support Behaviors Scale (Vaux et al., 1987).

Descriptive analyses were conducted to calculate frequencies for categorical variables and means and standard deviations for continuous variables. We summarized socio-demographic variables among participants in each grade level (4, 7, 10). Items for each outcome scale (e.g., Social Support Scale for Children) were summed to calculate total or subscale scores for each measure.

#### **Findings on Prevalence and Reporting**

The quantitative findings in the larger study (Mishna et al., 2015) show that rates of cyber witnessing were higher than cyberbullying and victimization at each assessment. In year 1, 24.2 percent reported cyber witnessing, 10.7 percent cyber victimization, and 2.9 percent cyberbullying. In year 2, 21.5 percent reported cyber witnessing, 7.6 percent cyber victimization, and 1.6 percent cyberbullying. In year 3, 25.1 percent reported cyber witnessing, 10.8 percent cyber victimization, and 2.5 percent cyberbullying. Similarly, rates of witnessing traditional bullying were higher than perpetration and victimization at each assessment. In year 1, 53.0% reported witnessing traditional bullying, 23.5% victimization, and 7.8% perpetration. In year 2, 42.6% reported witnessing traditional bullying, 17.3% victimization, and 4.3% perpetration. In year 3, 35.7% reported witnessing traditional bullying, 19.2% victimization, and 5.4% perpetration (Mishna et al., 2015). Of note, nearly half of all students (48.3%), who reported cyberbullying involvement in our survey, reported that they had not told an adult about what was happening online (Mishna et al., 2015). Moreover, 69.5% of students reported that cyberbullying and physical bullying are equally serious, and 64.5% believed that cyberbullying and “real” life verbal bullying are also equally serious (Mishna et al., 2015). These quantitative results serve as a springboard for the following discussion of qualitative findings, demonstrating that qualitative interviews reveal nuanced similarities and differences in the views of adults and youth, elucidating important interconnections among the levels of the ecological system (Mishna et al., 2004, 2009; Dennehy et al., 2020).

#### Qualitative Interview Data Collection and Analysis

Student participants in 4th grade (*n* = 20), 7th grade (*n* = 21), and 10th grade (*n* = 16) in the qualitative sub-sample were purposively selected for interviews from the larger quantitative sample, based on gender, grade, school need level, and whether they reported bullying/cyberbullying victimization, perpetration, or witnessing. After selecting student participants, their teachers (*n* = 30) and parents (*n* = 50) were invited to participate in interviews. Interviews lasted approximately 1 h, ranging in length from 30 to 90 min. All year 1 interviews (with students, parents, and teachers) took place in the school setting and utilized a semi-structured interview guide. Following preliminary analysis, this interview guide was refined for use in the year 3 follow-up phone interviews with the students and parents. Areas explored with students comprised understanding of cyberbullying and how it compares with traditional bullying, experiences of online aggression, and others’ attitudes and responses. Questions were informed by existing literature and the research team’s considerable experience. Parent and teacher interviews included questions on their awareness and understanding of cyberbullying, their child or student’s involvement in cyberbullying, links between cyber and traditional bullying, support, and their responses to cyberbullying.

Using a grounded theory inquiry, data were concurrently analyzed and theorized through constant comparison (Birks & Mills, 2015; Corbin & Strauss, 2008). Through this iterative process, the team used initial interview data and theoretical categories to inform and refine subsequent interview guides and data collection (Charmaz, 2014). The team members individually coded a portion of interviews to establish preliminary analytic focuses and inductively identify preliminary themes. Consistent with a grounded theory approach, no hypotheses guided data analysis and coders sought to bracket their biases through reflexive journaling and team discussions of assumptions (Corbin & Strauss, 2008). During team meetings, each interview was collectively coded, building upon, revising, and/or removing codes proposed by the initial coder. Emerging categories were developed and expanded. Axial coding promoted connections within and between categories and subcategories and enabled synthesis and explanation (Birks & Mills, 2015; Charmaz, 2014; Corbin & Strauss, 2008). Numerous preliminary codes were identified based on emerging themes that were generated and discussed. A holistic “middle-order” approach to coding resulted in a condensed number of initial codes (Saldaña, 2015). Axial coding was then used to identify connections within and between themes and subthemes (Birks & Mills, 2015; Charmaz, 2006, 2014; Corbin & Strauss, 2008). Through this iterative process of open, holistic, and focused coding, key themes emerged related to the understanding of traditional and cyberbullying according to the perspectives of the students, parents, and teachers. Measures were employed to ensure trustworthiness and authenticity. Prolonged engagement over the 3 years of the study ensured thick descriptions of the youth and adult narratives (Lietz & Zayas, 2010). Rigor was established through documentation for auditing purposes (Padgett, 2008). Trustworthiness and transferability were further ensured through reflexive journaling, bracketing, and dense descriptions (Corbin & Strauss, 2008).

While we use examples from our published manuscripts derived from our study entitled, “Motivations of Cyberbullying,” in the current manuscript, we identify new thematic areas and demonstrate how our qualitative interviews complement our quantitative findings. In analyzing findings across the study publications and datasets, we have not previously drawn the conclusions. The unique contribution of the current manuscript is the use of findings of previous publications to generate broader conclusions about the benefits of a mixed-methods approach (qualitative interviews and quantitative survey data) that makes visible the connections across ecological systems levels.

In discussing how qualitative research contributes to understanding bullying and cyberbullying and complements quantitative findings, the following new thematic areas are discussed: augmenting quantitative findings through qualitative interviews, contextualizing new or rapidly evolving areas of research, capturing nuances and complexity of perspectives, and providing moments for self-reflection and opportunities for learning.

### Augmenting Quantitative Findings Through Qualitative Interviews

By examining process, context, and meaning for participants, qualitative methodology can augment quantitative findings. Quantitative methodology establishes outcomes and causal relationships and puts forth generalization and predictions (Yilmaz, 2013). Our background study which was a longitudinal multi-informant mixed methods study (Tashakkori et al., 1998) used grounded theory (Strauss & Corbin, 1998) and a longitudinal quantitative design to aid understanding of nuances related to cyberbullying (Mishna et al., 2009). In creating opportunities for the voices of young people to be heard (Carroll & Twomey, 2020; Gilgun & Abrams, 2002), qualitative methodology is especially useful for phenomena that are largely unstudied and/or rapidly evolving, such as cyberbullying, by explicating process and a holistic understanding and directions for future research (Mishna & Van Wert, 2013; Gilgun & Abrams, 2002).

In our paper, “Benchmarks and bellwethers in cyberbullying: The relational process of telling”^3^ (Mishna et al., 2020), the qualitative analysis revealed relational processes among students that occurred when they considered whether to tell adults about their bullying and cyberbullying experiences. As noted above, almost half of the students who reported cyberbullying involvement relayed that they had not told an adult. Qualitative findings, however, exposed complex interactions that informed their decision-making processes. Reticent about speaking with adults, students turned to friends. It emerged that in addition to sharing, telling friends often served as a bellwether to gauge whether to proceed and report the situation to an adult. Often minimizing the severity of their ordeal, many students had decided against informing adults, frequently mentioning their concern about making a “big deal.” Participant interviews further revealed that media reports of high-profile cases involving cyberbullying can serve as benchmarks through which to assess the severity of their own personal experiences. The qualitative findings in our study helped to contextualize the quantitative data by unpacking and making visible the reasoning and contributing factors, thus increasing understanding of what informs youth’s decisions regarding whether and who to tell about cyberbullying involvement. By augmenting the quantitative data detailing the proportion of youth who do not tell adults, particulars attained through qualitative interview data help to inform and direct prevention and intervention strategies that are concrete and actionable for addressing the more challenging aspects of cyberbullying involvement and disclosure. In offering insights on the relational dynamics among peers and between youth and adults with respect to cyberbullying, the qualitative analysis gave voice to these interconnected layers of the youths’ ecological environment.

### Contextualizing New or Rapidly Evolving Areas of Research

While cyberbullying is no longer considered a new phenomenon, the rapid development of technology is continually altering the cyber landscape, creating a need for perpetual knowledge generation (Odgers & Jensen, 2020; Rosa et al., 2019) and for evolving definitions, measurements, and responses (Spears et al., 2009). Moreover, rapid and ongoing technological advances create unique challenges for practitioners, policy makers, and researchers, in remaining current and responding to cyberbullying (George & Odgers, 2015; Jäger et al., 2010). With youth at the forefront of technological advances in many ways, qualitative methodology is well suited to elicit the experiences and perspectives of young people in promoting in-depth understanding of youth cultures, dynamics, and processes (Thornberg & Knutsen, 2011).

The data collection for our background study occurred between 2012 and 2014, during the early stages of attention to and research on sexting (sending and receiving sexually explicit images, videos, and text among youth). In the quantitative questionnaires, we included one question related to sexting for students in grades 7 and 10 and their parents and teachers. Our quantitative survey found that 15.6% of students in grades 7 and 10 had seen nude or sexual photos of friends, family, boyfriend, girlfriend, or other romantic partner online or over a cell phone. Furthermore, 27.8% of teachers had witnessed or were aware of their students viewing sexually explicit images, video, or text on cell phones at school. The data indicated that digital sending and receiving of sexually explicit images, video, or text was a new phenomenon among youth participants in grades 7 and 10 in a rapidly changing digital environment.

We did not explicitly inquire about sexting in the interviews with students, parents, and teachers. Rather, we asked participants about the students’ negative experiences with cyber technology. During analysis of the interview data, however, sexting emerged as a new and pertinent phenomenon among youth, which generated knowledge about rapidly evolving cyber dynamics that warranted further attention and inspired a paper entitled, “Gendered and sexualized bullying and cyberbullying: Spotlighting girls and making boys invisible” (Mishna et al., 2021b). The qualitative interview data in this instance confirmed our quantitative findings on sexting among youth and allowed us to delve into the complex and nuanced ways participants articulated sexting behaviors along gender lines that both reinforced and were reinforced by gendered sociocultural norms and pressures. In student accounts, boys’ presence and participation in cyberbullying were frequently invisible, such as the non-consensual sharing of sexual images. Blamed for their poor choices, girls were spotlighted and their behavior problematized through negative characterizations. The participants’ focus on girls as responsible for the gendered cyberbullying and non-consensual sharing of images corresponds with how youth are typically educated about digital technologies through an “online safety model” with the focus on youth protecting themselves and avoiding “risky” activities (Johnson, 2015). As such, our findings provided context for this rapidly evolving environment that then allowed us to draw links between individual cyberbullying behaviors, understanding and articulation of these behaviors, and the broader influence of patriarchal structures (Mishna et al., 2021b). The qualitative findings underscored the need to consider key factors that go beyond individual characteristics and behaviors and to develop education and prevention and intervention strategies that address sociocultural norms and values. The qualitative findings stimulated new research endeavors and collaborations with community organizations and academics.

### Capturing Nuances and Complexity of Perspectives

Bullying and cyberbullying are exceedingly complex and must be studied within the contexts of the involved youth as well as within the larger social context of youth (Cross et al., 2015; Dennehy et al., 2020; Johnson & Puplampu, 2008; Sainju, 2020; Thornberg, 2011). An ecological systems framework is appropriate as it provides insight into the interconnected relationships among varying aspects and social layers of an individual’s world (Bronfenbrenner, 1979). While quantitative research considers and articulates context, qualitative interviews provide an occasion to engage with the richness of students’ perspectives, thoughts, and feelings about themselves and their social worlds (Mishna et al., 2004) and allow for a deeper understanding of youth culture and social processes from the vantage point of young people (Chaumba, 2013; Dennehy et al., 2020; Spears et al., 2009; Thornberg & Knutsen, 2011). Although qualitative studies are generally bound by a particular timeframe, participants bring their life histories and cumulative experiences to the research engagement (Phoenix et al., 2003), which can generate a fulsome and holistic understanding of cyberbullying, taking into consideration individual, family, peer, school, cyber, and sociocultural conditions over time.

Qualitative interview data allow for an interpretive approach that draws upon patterns of understanding, similarity, and contradiction, thereby teasing out underlying assumptions that shape how people define and assess experiences and phenomena such as bullying and cyberbullying (Mishna et al., 2020, 2021a). In our paper entitled “Looking Beyond Assumptions to Understand Relationship Dynamics in Bullying”^4^ (Mishna et al., 2021a), analysis of the qualitative interview data exposed persistent and pervasive assumptions about bullying linked to sociocultural norms and understanding of gender. These assumptions shaped participants’ understanding and conclusions of bullying and cyberbullying experiences, behavior, and motivations. Focusing on the visible hurt and injuries associated with physical bullying, participants tended to make comments such as “you’ll heal in a few days,” whereas they noted that with verbal bullying, the mental anguish “might stay for a long term.” This viewpoint that physical bullying was not a relationship problem appeared to be linked to gender stereotypes and social norms regarding the “natural” behavior of girls and boys. These gendered assumptions led participants to suggest that addressing bullying among girls was “complicated” and ongoing, whereas addressing physical bullying among boys was “simpler” and faster, a finding similar to that of Eriksen and Lyng (2018) who described participants’ descriptions of bullying among boys as “undramatic.” These assumptions appeared to preclude participants from discussing physical bullying among boys in a manner that acknowledged the physical bullying involvement as entrenched in relationship dynamics.

Qualitative interviewing provides an opportunity for participants to express their views and ideas when discussing the topic of interest which can elicit novel conclusions and nuances. As an example, at times, youth who claimed not to have involvement with cyberbullying may go on to describe situations that actually seemed to fit the definition of cyberbullying. In our Spotlighting Girls paper, many participant reports aligned with stereotypes regarding differences in how boys and girls bully others. These stereotypes were shared, however, even when they contradicted participants’ own experiences. For instance, similar to other research findings (Eriksen & Lyng, 2018), one participant described a boy as using “guilt trips” as a bullying tactic, yet described boys as only bullying physically. Consequently, relational aggression among boys often goes unnoticed and remains invisible. Similarly, the same behavior displayed by both girls and boys was discounted in boys and highlighted in girls. Boys’ behaviors were often not considered to be bullying because they were positioned as within the bounds of masculine gender norms. For example, one girl reported that “mostly girls, not boys,” bully “because boys would just go over and do some physical things... [Girls would] post embarrassing stuff about the person and do that kind of stuff” (p. 410). It is possible therefore that such actions by boys were not identified as bullying and thus underreported in the quantitative surveys while captured in the interviews. Discrepancies emerged in how cyberbullying had been reported in quantitative measures and how it was described in the interviews. This indicates that qualitative interviews can complement quantitative findings by revealing the complexities and ramifications of social experiences which are not reported in quantitative surveys.

The critical role of witnessing in bullying and cyberbullying is well documented (Salmivalli, 2010, 2014; Spadafora et al., 2020; Volk et al., 2014). Social experiences related to witnessing are also complex, and bystander decision-making and responses impact both the process and outcomes of bullying incidents (Salmivalli et al., 2011). Qualitative research can offer youth the opportunity to explore and explain the motivations and factors they consider in determining whether to intervene, specifically the social costs and benefits of intervening (Spadafora et al., 2020). Our qualitative interviews similarly added youth voices concerning the dilemmas they faced in considering whether and how to respond based on emotional and contextual factors (Mishna et al., 2021b), thus providing nuanced perspectives that serve to augment the quantitative findings related to bystander responses.

### Providing Moments for Self-reflection and Opportunities for Learning

Qualitative methodologies are recognized as providing participants opportunities to self-reflect in the context of being listened to empathically (Birch & Miller, 2000; Wolgemuth et al., 2015). According to a systematic review of quantitative, qualitative, and mixed-methods studies conducted with children and adolescents, participation was mainly considered to be beneficial (Crane & Broome, 2017). Negative responses to participating in the research included feeling anxious and upset (Crane & Broome, 2017). Research indicates that despite describing negative effects of participating, children and youth reported that overall it was more positive to participate in the research (Crane & Broome, 2017) or described the emotional pain they experienced as beneficial in various ways, for example, as “emotionally cleansing” (Wolgemuth et al., 2015, p. 366). The qualitative research process offers participants the opportunity to come to new understandings and can reveal evolving thoughts within participant narratives (Birch & Miller, 2000; Wolgemuth et al., 2015). Qualitative processes are iterative and involve probing questions that can prompt dynamic reflection by participants (Wolgemuth et al., 2015). Birch and Miller (2000) explain that they “use the term therapeutic to represent a process by which an individual reflects on, and comes to understand previous experiences in different—sometimes more positive—ways that promote a changed sense of self” (p. 190).

Recognizing the potential risks in research with children and youth (Mishna et al., 2004; Crane & Broome, 2017), we informed the students in our study of the possible risks should they decide to participate, such as the possibility that they would become upset as we were asking them about hurtful matters, and the limits to confidentiality. Anticipating that some of the questions could lead to a participant becoming distressed or disclosing potentially sensitive or upsetting information, we put in place a protocol (approved by the university and school board research ethics boards) to identify and offer support for students in distress (Mishna et al., 2016).

Corresponding with previous research, the reflexivity of sharing their narratives and views seemed to contribute to some participants coming to a different understanding of their experiences. Such reflection was evident in our interviews with students and their parents and teachers. When asked whether he had witnessed cyberbullying, for example, a boy reflected that only in being asked about cyberbullying in the interview did he recognize the behavior as cyberbullying: “When I think about it now, I actually did a few times. I didn’t feel that it’s cyber bullying, I wasn’t thinking that it’s a huge deal. It’s basically a few arguments between people on Facebook, like writing things about each other in public, not in private, chats.”

In another example, a parent reconsidered her views during the interview. This parent first commented that girls and women are “more vindictive” than boys and men, who, she explained, have “your spat, you get over it, and you move on.” After reflecting on her assumptions, she wondered how much of this widely held view of the behavior “is just media driven because I guess the victims that we see on the news, at least in Canada, have been girls, right?… but that doesn’t say that boys aren’t also being bullied.” Similarly, a girl contemplated her assumptions after first casting boys in a favorable light in contrast to girls. In commenting that girls bully each other because of appearance, she praised boys, “because usually they don’t tend to worry about those things...They’re proud of themselves, and they don’t pick on other people. They’re good with what they have.” After pondering these stated differences between boys and girls, this girl surmised, “I think it’s from when we were little because those Barbie dolls are super skinny. We wanted to have blonde hair, blue eyes, and be like Barbie. I think it’s just how maybe we were raised.” Another girl, who asserted that while cyberbullying occurred with equal frequency among boys and girls, added that it was not “a big thing” for boys, in contrast to girls who, “would show it off more, be like oh yah, blah, blah, blah.” Rather than concluding that this difference indicated that cyberbullying was not a big deal for boys, however, this girl attributed the difference between boys and girls to dominant masculinity norms. She asserted that “guys kind of hide it in more” and explained that “they don’t want to show that they’re weak because guys tend to be, they think that they’re very strong, kind of thing.” The evolving perspectives throughout this and the previous exchanges demonstrate the process of deepened understanding that can occur because of qualitative interviewing.

Such new understanding can inspire a desire to act and make change through community engagement. A girl explained that the research was the first time she had spoken with anyone about cyberbullying. This girl’s appraisal of her participation is consistent with findings in which participants may be motivated to take part in research for the opportunity to effect and advocate for change and help others (Cutcliffe & Ramcharan, 2002; Wolgemuth et al., 2015). She remarked that participating had been a helpful process which led her to,

think of different ways that I could help someone else if I see it happening… Just talking about it makes you think about what could cause it, what could make someone bully someone else. It makes you realize how it could make someone feel. Also, talking about how there isn’t really a support system at school. It makes me want to go and talk to someone to organize it, because it does happen a lot and I know it affects a lot of people

## Conclusion

The inclusion of qualitative interviews in mixed methods research brings forth new information about content, process, and meaning that is otherwise not visible. By engaging youth voices as well as adult perspectives through both quantitative measures and qualitative interviews in the mixed methods study discussed in this manuscript, entitled Motivations for Cyberbullying, understanding of bullying and cyberbullying was advanced, thus enriching the quantitative methodology. The findings of the interviews extended knowledge related to bullying and cyberbullying in the following ways, which can inform “bottom-up research and intervention efforts” (Dennehy et al., 2020, p. 10): augmenting quantitative findings, contextualizing new or rapidly evolving areas of research, capturing nuances and complexity of perspectives, and providing moments for self-reflection and opportunities for learning.

Qualitative research constitutes a significant venue through which to amplify the voices of children and youth (Dennehy et al., 2020) and ensures that children and youth’s experiences of the world are represented in understanding social phenomena (Mishna et al., 2004; Carroll & Twomey, 2020; Chaumba, 2013; Dennehy et al., 2020; Patton et al., 2017). According to Dennehy and colleagues (2020), engaging youth as co-researchers in cyberbullying research may enhance efforts to ethically and earnestly amplify youth voices. A synthesis by Elsaesser et al. (2017) supports the view that focusing on collaboratively working with youth to understand and safely navigate the cyber world through education and empowerment is more effective than interventions aimed at restricting ICT use without involving youth. Through quantitative measures and qualitative interviews, our mixed methods study examined participant perspectives regarding bullying and cyberbullying on the various ecological systems levels across the students’ lives. The use of mixed methods facilitated a dialogue between the participant responses to both methodologies, thus highlighting the salience of the overlapping influence and interactions among the systems levels. Such complex and nuanced understanding is necessary to inform meaningful prevention and intervention strategies to address bullying and cyberbullying.

According to the United Nations Convention on the Rights of the Child (Assembly UG, 1989), children and youth have the right to discuss their views and experiences. The Convention states that all children have the right to protections, provisions, participation, and non-discrimination (Assembly UG, 1989). Participation entails the right for children to express themselves and have a voice in situations that have to do with and affect them. The importance of listening to children’s voices underscores the limits of adult proxies in representing children’s emotional and social worlds (O’Farrelly, 2021). Bullying and cyberbullying fundamentally violate these protections, silence children’s voices, and compromise their healthy development (Greene, 2006). Our mixed methods study through quantitative measures and qualitative interviews facilitated a dialogue between the participant responses in both methodologies. This interaction of data types maximizes the voices of and collaboration with participants as well as knowledge generation.

## Data Availability

Not applicable.

## Footnotes

1 Different terms are used to describe the same approach (e.g., social-ecological framework, ecological systems framework, ecological theory, ecological perspectives). For the purposes of this paper, the term ecological systems framework is used.

2 All additional references to this research study will be shortened to “Motivations for Cyberbullying.”

3 All additional references to this paper will be shortened to “Benchmarks and Bellwethers paper.”

4 All additional references to the paper will be shortened to “Relationship Dynamics paper.”
